# Composition, Microstructure, and Electrical Properties Control of the Powders Synthesized by Sol–Gel Auto-Combustion Method Using Citric Acid as the Fuel

**DOI:** 10.1186/s11671-017-1976-1

**Published:** 2017-03-31

**Authors:** Bogdan K. Ostafiychuk, Larysa S. Kaykan, Julia S. Kaykan, Bogdan Ya. Deputat, Olena V. Shevchuk

**Affiliations:** 1 Vasyl Stefanyk PreCarpathian National University, 57 Shevchenko Str., Ivano-Frankivsk, 76018 Ukraine; 2 G.V. Kurdyumov Institute for Metal Physics, N.A.S. of Ukraine, 36 Academician Vernadsky Boulevard, UA-03680 Kyiv-142, Ukraine; 3 Ivano-Frankivsk National Technical University of Oil and Gas, 15, Karpatska Street, 76019 Ivano-Frankivsk, Ukraine

**Keywords:** Nanosized ferrite, Impedance, Dielectric constant, Conductivity, 71.20.Nr, 72.15.Eb, 72.20.Pa, 77.22.Gm, 73.22.-f, 76.80. + y

## Abstract

Nanocrystalline lithium ferrite *Li*_0.5_ *Fe*_1.7_ *Mg*_0.8_ *O*_4_ powders were synthesized by the sol–gel auto-combustion method from the corresponding metal nitrates using citric acid as fuel.

The results from XRD, SEM, and AC electrical conductivity studies are summarized as follows:

- The results of XRD analysis showed that all the samples were formed in single-phase cubic spinel structure at different annealing temperatures from 300 to 700 °C for 2 h. The lattice parameter was found to decrease on increasing the temperature.
- The microstructure of lithium ferrite powders was temperature dependent. The particle size was increased with the annealing temperature.
- AC electrical properties were investigated using the super-linear power law and activation energies were calculated for all compositions. The electron mobility in *Li*_0.5_ *Fe*_1.7_ *Mg*_0.8_ *O*_4_ samples ranged from 0.05 to 0.29 eV, which clearly indicated that the present lithium ferrites have semiconductor-like behavior.
- The frequency exponent “s” of lithium ferrite lies in the range 0.5 < *s* < 1, which confirms the electron hopping between *Fe* ^2 +^ and *Fe* ^3 +^ ions.

## Background

Spinel-type ferrite nanomaterials evoke increased interest due to their high optical, structural, electrical, and magnetic properties compared to their microcrystalline analogs. Such properties of ferrite nanoparticles promote their wide use in various fields of technology and medicine, nanocrystalline magnetic materials, in particular, and can be used as substitutes for pure metals as permanent magnets in some devices because of their high resistance, low eddy current loss, and low magnetic losses at relatively low cost [1]. According to its crystal structure, ferrite-spinel has cubic close-packed arrangement of oxygen lattice with two types of sites: tetrahedral (A) and octahedral (B). Depending on the composition and distribution in two cationic sub-lattices, such nanocrystalline spinel can detect different magnetic and dielectric properties. At a certain substitution and cation distribution of dopant ions on ferrite-spinel, sub-lattice can detect ferromagnetic, antiferromagnetic, spin-cluster, or paramagnetic properties [2]. Properties of ferrites, including electrical, largely depend on their microstructure, which is determined by the degree of dispersion of the material, sintering temperature, time, and conditions of synthesis and composition. The microstructure of the material is formed during sintering and is determined by the size of crystallites, their shape, porosity, degree of agglomeration, chemical, and phase composition, which in turn depends on the technology and synthesis conditions [3].

The traditional method of obtaining ferrites is solid phase synthesis (ceramic sintering technology dual) [4]. However, the traditional ceramic method has several disadvantages such as the problem of purity control, chemical heterogeneity, large size of the particles, and additives are added at grinding which is also necessary for the synthesis at high temperature (> 1000 °C). The alternative is chemical synthesis method, in which the size of the synthesized particles does not exceed the nanometer range, does not require high temperatures during synthesis, and provides high phase and chemical homogeneity of the final product. One of the most effective methods of synthesis is sol–gel auto-combustion method [5], in which the precursor is decomposed at temperatures much lower than 500 °C, and the heat required to form the final product is released by exothermic redox reactions.

The purpose of this work is to obtain nano-powder particles of magnesium-substituted lithium ferrite by sol–gel auto combustion using citric acid as the fuel, and to research the effect of synthesis conditions and the concentration of replacing elements at the phase of composition, morphology, and electrical properties of the material.

## Methods

Magnesium-substituted lithium ferrites were synthesized in the following way: aqueous solutions of metal nitrates (Fe(NO_3_)_3_ · 9H_2_O, LiNO_3_ · 3H_2_O, Mg(NO_3_)_2_ · 3H_2_O) were used as stock reagents, taken in the appropriate molar ratio by the expected stoichiometric of compounds and citric acid. Nitrates of metals and citric acid were mixed drop by drop in the magnetic mixer in the molar ratio of metal to citric acid of 1: 1. Metal nitrate solution—citric acid—slowly evaporated in the oven to form a viscous gel. The following drying was performed for the complete removal of adsorbed water at 110 °C. The resulting gels were placed in an oven that was heated to a temperature of about 200–220 °C then mixture flashed and the reaction (1) formed the final product.

1 $$ \begin{array}{l}0,5{\mathrm{Li}\mathrm{NO}}_3+2,5- x\mathrm{Fe}{\left({\mathrm{N}\mathrm{O}}_3\right)}_3\cdot 9{\mathrm{H}}_2\mathrm{O}+ xMg( N{O}_3){}_2\cdot 6{H}_2 O+ n{\mathrm{C}}_6{\mathrm{H}}_8{\mathrm{O}}_7\to \\ {}\to {\mathrm{Li}}_{0,5}{\mathrm{Fe}}_{2,5{\textstyle \hbox{-}}\mathrm{x}}{\mathrm{Mg}}_{\mathrm{x}}{\mathrm{O}}_4+4{\mathrm{N}}_2+6 n{\mathrm{C}\mathrm{O}}_2+\left(22,5+4 n\right){\mathrm{H}}_2\mathrm{O}+\left(10-4,5 n\right){\mathrm{O}}_2.\end{array} $$

**Fig. 1:**
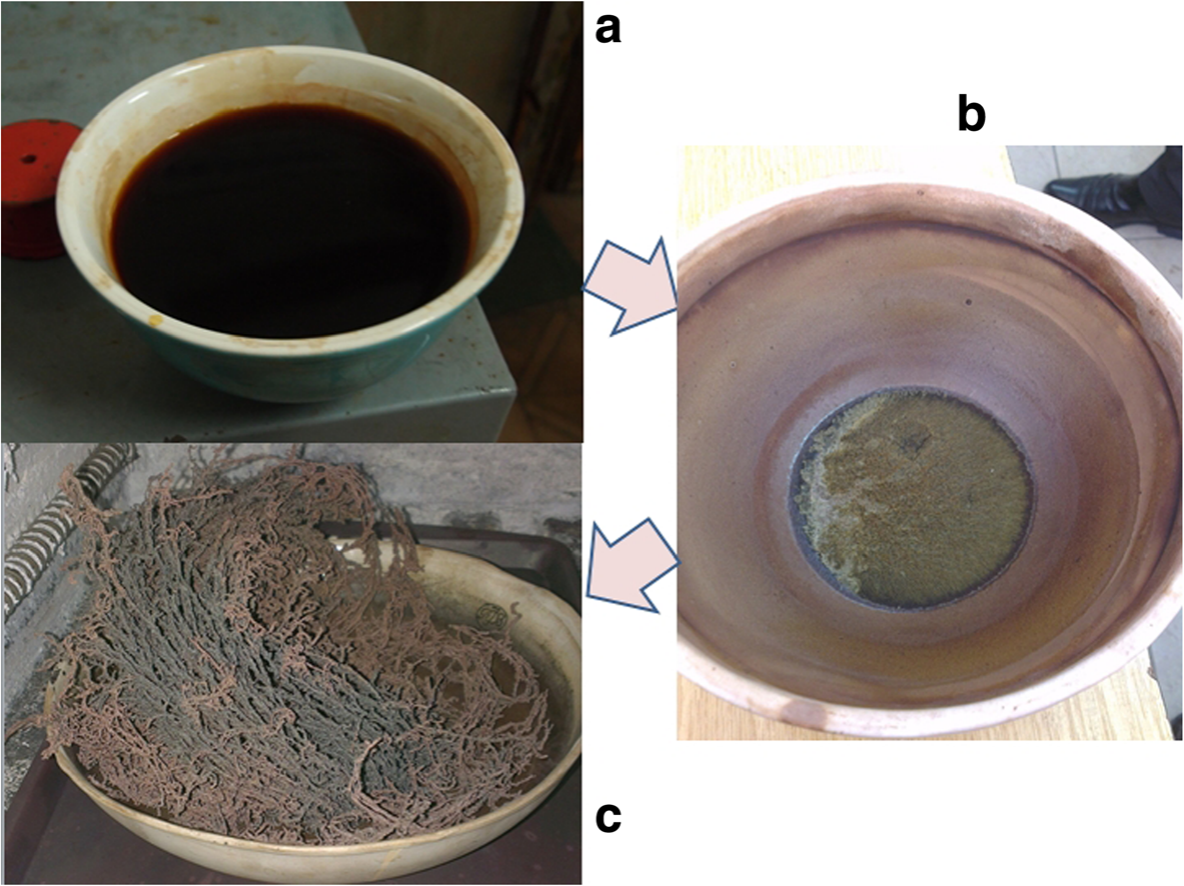
The procedure for obtaining nanodispersed magnesium-substituted lithium ferrite by sol–gel auto combustion. **a** Precursor solution. **b** The dried gel the powder obtained after auto combustion

More detailed information about this synthesis method is given in [6] (Fig. 1).

X-ray diffraction spectra were obtained on a diffractometer DRON-3 in CuKα-radiation (*λ* = 0.1540598 nm) in the range of angles 20 < 2θ < 65^0^ within a step of scan 0.05^0^ in Bragg-Brentano geometry. The dimensions of CRD synthesized product were determined by two methods: by Scherrer, $\left\langle D\right\rangle =\frac{\lambda}{\beta_{1/2} \cos \theta}$, where *λ* is the wavelength of X-ray, θ is the diffraction angle, β_1/2_ is half-width of reflection, and also by interpolation method of Williamson Hall, in accordance with βcosθ from sinθ of the equation $\beta \cos \theta =\frac{\lambda}{D}+4\varepsilon \sin \theta$ (if the approach was performed with Lorentz or Cauchy functions) or of the equation ${\beta}^2{ \cos}^2\theta ={\left(\frac{\lambda}{D}\right)}^2+{\left(4\varepsilon \sin \theta \right)}^2$ (if it was carried out by Gauss function).

The image of transmission electron microscopy, high-resolution (HRTEM) were obtained with the use of a microscope FEI Tecnai Orisis TEM/STEM 80–200 at a 200 kV.

The Mössbauer spectra were measured with a MS-1104Em spectrometer using a 57Co γ-ray source and calibrated at room temperature with α-Fe as a standard (line width 0.29 mm/s). The isomer shifts (δ) are relative to Fe metal. The model fitting was performed using Mosswin 3.0 software.

To conduct research on impedance synthesized powder, there were tablets shaped in a diameter of 18 mm and thickness of 0.6 mm after pressure being dried at 65 °C for 5 h. The tablets were used to create a capacitor system graphite electrode/sample/graphite electrode. Conductivity at alternating currents, tangent loss, real, and imaginary part of permittivity was determined on the basis of experimental dependencies of complex impedance obtained at Autolab PGSTAT 12/FRA-2 spectrometer at frequencies 0.01Hz–100 kHz. The temperature curves were derived from research mode of impedance steps while heating with isothermal holding after every 50° in the temperature range 295–723 K.

## Results and discussion

### Structural Studies

**Fig. 2:**
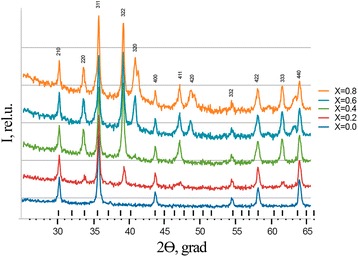
X-ray experimental spectra of synthesized samples Li_0.5_Fe_2.5-x_Mg_x_O_4_

**Fig. 3:**
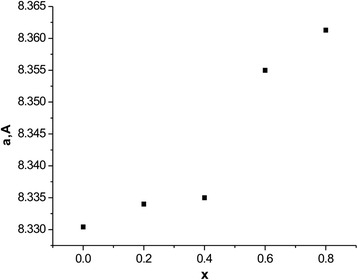
The dependence of the stable lattice on synthesized samples of the composition

The resulting system is a single-phase spinel space group Fd3m as it is understood by reflexes (220); (311); (400); (511); (440); and (422) (Fig. 2). There were no additional phases of corresponding nitrates or oxides of initial components found. It confirms that the reaction was complete and there was not any other component left (Fig. 3).

Cation distribution obtained from the analysis of experimental X-ray diffraction method using full profile Ritveld and calculated values of lattice constants are shown in Table 1. The calculated values of ionic radii oxygen and theoretical optional value of lattice constants for each composition are shown in Table 2. It shows that the experimental mentioned lattice constants are slightly bigger than theoretically calculated. Obviously, this increase can be explained by the influence of the surface that appears when the crystallite size does not exceed 100 nm.

**Table 1:** Cation distribution of synthesized samples Li_0.5_Fe_2.5-x_Mg_x_O_4_

*x*	A-position	B-position	*a*, Å	Δ*a*, Å
0.0	Fe_1.0_	Li_0.5_Fe_1.41_	8.330	±0.002
0.2	Mg_0.16_Fe_0.92_	Li_0.5_Fe_1.38_Mg_0.04_	8.334	±0.002
0.4	Mg_0.32_Fe_0.84_	Li_0.5_Fe_1.26_Mg_0.08_	8.335	±0.002
0.6	Mg_0.45_Fe_0.76_	Li_0.5_Fe_1.14_Mg_0.12_	8.355	±0.002
0.8	Mg_0.64_Fe_0.68_	Li_0.5_Fe_1.02_Mg_0.16_	8.361	±0.002

**Table 2:** Values of ionic radii and theoretical value of lattice constant

*x*	*a*_exp_, Å	*a*_th_, Å	r_B_, Å	r_A_, Å	r, Å	u, Å
0.0	8.330	8.208	0.640	0.626	0.633	0.2600
0.2	8.334	8.295	0.691	0.629	0.660	0.2604
0.4	8.335	8.305	0.742	0.604	0.673	0.2608
0.6	8.355	8.316	0.794	0.578	0.686	0.2612
0.8	8.361	8.327	0.845	0.553	0.699	0.2616

Cationic distribution (Table 1) shows that cations Li^+^ occupied only B positions, whereas Fe^3+^ and Mg^2+^ ions occupy both A- and B positions in sub-lattice. Iron ions are redistributed by A and B sub-lattices in a ratio of about 4:6 and magnesium ions 8:2, respectively. According to the results of X-ray diffraction, there is a definite preference in position of these ions Li^+^ > Fe^3+^ > Mg^2+^.

Table 3 shows the values of calculation for CRD by two methods for all synthesized systems.

**Table 3:** CRD of synthesized samples Li_0.5_Fe_2.5-x_Mg_x_O_4_

*x*	CRD, nm	CRD, nm
	Sherrer method	Williamson-Hall method
0.0	42	40
0.2	38	35
0.4	37	35
0.6	20	19
0.8	20	18

**Fig. 4:**
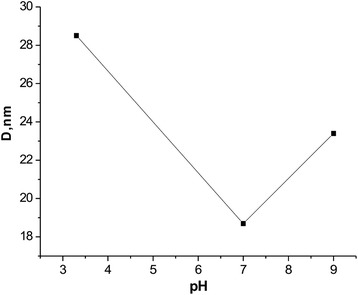
CRD system of dependence on the pH of the reaction medium

To determine the influence of synthesis conditions on morphology of obtained materials, synthesis was carried out at different pH values within the reaction medium (pH = 3, 7, and 9). The dependence size of CRD from pH of the reaction medium is shown in Fig. 4.

Thus, the effect of pH of the reaction medium on the microstructure of synthesized materials can be interpreted as follows: to establish the proper pH of the solution precursors there was aqueous ammonia solution (NH_4_OH) added. Ammonia increases the forming gel of metal cations of citrate, which helps to control the oxygen balance and promotes the formation of three-dimensional porous (3D) structure nitrate citrate gels [7]. At low pH values, there is insufficient process of gel formation of metal ions with citrate and this leads to an imperfect 3D structure of the gel and thus nitrate oxidant implemented in gels is easily decomposed in the process of drying and during autocombustion. The result will decrease the amount of heat generated due to the shortage of oxidant and the resulting product will have a relatively dense structure. If ferrite size is about 20 nm CRD, the optimum pH is 7. The decomposed of citric acid contained in photocopying gels occurs at 220 °C and as a result, a single phase spinel ferrite is formed. The decomposed of citrate-nitrate gels completely without ammonia occurs only at 500 °C. Gels obtained from solution at pH > 7 prevent the process of spontaneous ignition, so even when forming a single-phase structure of ferrite, its morphology is quite dense and as a result, a system characterized by high values of CRD formed. When pH = 3, gel reveals a dense network microstructure in which there are only single pores in the structure. If pH is increased, there is gel developed network. At pH = 6 and 7 3D network structure is fully formed. However, such a porous net structure makes it possible to add more oxygen into xerogels. Oxygen accelerates the combustion process, thus combustion temperature and speed are increasing. Porous structure makes burning gels fast and very strong. Moreover, the NH_4_NO_3_ decomposition accompanied by O_2_ release accelerates the combustion process.

**Fig. 5:**
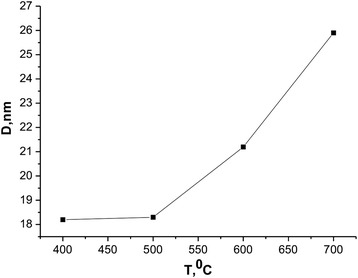
Dependence size of CRD temperature annealing

In order to establish the effect of temperature on the structure of the synthesized material, we conducted post synthesis annealing at temperatures of 300–700 °C. As it is seen in Fig. 5, a significant increase in the size of crystallites starts at temperatures >500 °C. By choosing the appropriate pH of the reaction medium and post synthesis temperature annealing can be directed to form a material with the desired morphology [8].

**Fig. 6:**
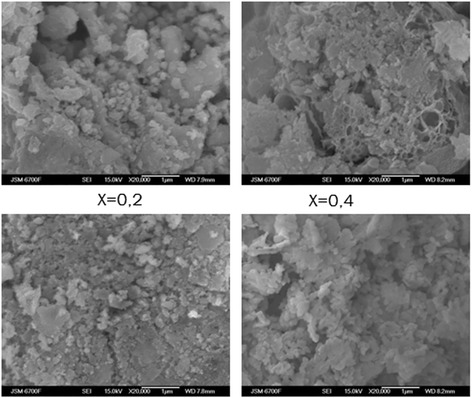
Micrographs of the systems Li_0.5_Fe_2.5-x_Mg_x_O_4_

In Fig. 6, there are micrographs of the synthesized systems.

As you can see from the image, microstructure of uniformly distributed system is characterized by small grains separated by borders. The average particle size of ferrite compositions derived from images stored SEM is in the vicinity of 78–87 nm. The average size of ferrite grains synthesized obtained by SEM is larger than the size of particles obtained by XRD. This can be explained by the fact that every grain is the result of the agglomeration of several nanocrystals.

**Fig. 7:**
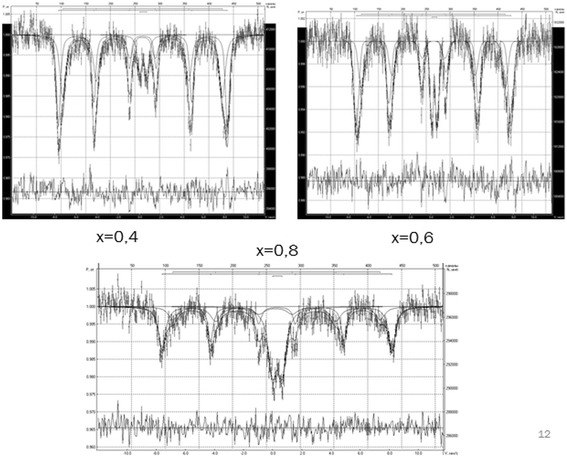
Mossbauer spectra of system Li_0.5_Fe_2.5-x_Mg_x_O_4_

Mössbauer spectra ^57^Fe of synthesized systems obtained at room temperature are presented in Fig. 7.

The whole received spectrum is a superposition of two sextets of corresponding tetrahedral and octahedral sub-lattices of ferrite spinel. In addition to the visible spectrum, there is paramagnetic doublet with quadruple splitting ~0.67 mm/s. The emergence of a doublet with sextets can be explained as follows: major changes or over-changeable interaction of magnetic neighbors are responsible for magnetic ordering. In the researched system, the replacing magnetic atoms included in the exchange interaction with this atom of iron to nonmagnetic Mg^2+^ ions isolates some of the other iron atom of the lattice of magnetic ions involved in magnetic interactions. The options that decode mössbauer spectra are shown in Table 4.

**Table 4:** Parameters of Mossbauer spectra

Sample	Sextet A					Sextet B					Doublet			
	S, %	H_eff_, kOe	δ, mm/s	Δ, mm/s	Γ, mm/s	S, %	H_eff_, kOe	δ, mm/s	Δ, mm/s	Γ, mm/s	S, %	δ, mm/s	Δ, mm/s	Γ, mm/s
*x* = 0.4	42.07	493.0	0.31	0.01	0.45	38.31	465.1	0.29	−0.02	0.69	19.62	0.34	0.70	0.82
*x* = 0.6	23.73	490.7	0.29	0.02	0.47	34.82	447.51	0.29	0.08	0.34	41.44	0.31	0.64	0.79
*x* = 0.8	51.34	466.0	0.28	−0.01	0.59	32.30	433.07	0.26	−0.03	0.67	16.36	0.36	0.53	0.39

*S* the area under the spectrum, *H*_*eff*_ a magnetic field in the core, *δ* isomer shift, Δ quadrupole splitting, *Γ* line width

### Electrical Properties

**Fig. 8:**
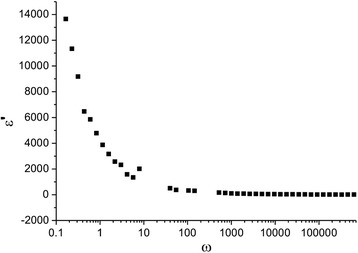
Dependence of real part of dielectric constant from the frequency at 273 K

Figure 8 shows the change in dielectric constant (ε´) as a function of frequency from 0.01 to 10^5^ Hz. In the research, frequency range of the system revealed a strong dielectric dispersion. This means that at low frequencies the dielectric constant is high, and with the increase of frequency and decrease of its value there is almost a stable value at frequencies >100 Hz.

Dielectric dispersion in general can be described basing on four fundamental polarization mechanisms that exist in materials [5]. It is electronic, ionic, or dipolar and in between the borders polarization. In the applied electric field, the displacement of an electron from the atom nucleus causes electronic polarization, while the increase of the ionic separation between positive and negative ions in the ion type of connection provides ionic polarization. These two polarizations usually occur at very high frequencies in the 1 GHz^−1^ THz. Accordingly, the trend orientation of dipoles along the field direction causes the growth of dipolar polarization, while the accumulation of charge on the electrode surface or the interface in a multiphase material gives the rise to space charge or in between field polarization. However, since the frequency range in this research is limited to 10^5^ Hz and the compounds have uniform fine microstructure with fine grain boundaries and the possibility of coexistence of significant number, the impact to dielectric polarization is done due to dipolar and in between the borders polarization. The increase of the low dielectric constant is provided due to the influence of the electronic exchange that occurs in ferrite as a result of local displacement of electrons in the direction of the applied electric field *Fe* ^2 +^ ⇔ *Fe* ^3 +^ [5]. In addition, since the microstructure of the material is heterogeneous, composed of grains and grain boundaries, their dielectric behavior also contributes to in between polarization. Another factor that affects the processes of accumulation of charges on inter-grain borders is a partial loss of lithium ions and oxygen that can occur during the synthesis and subsequent annealing. As a result, the possible formation of lithium oxygen vacancies that migrate towards the surface and accumulate at the grain boundaries inter-grain and thus contribute to the increase in low polarization. Thus, the strong growth of the dielectric constant at low frequencies is caused mainly by two mechanisms, namely dipole-orientation caused by local displacement of electrons under the influence of the applied field and in between one. Within increasing of temperature frequency, the independent dielectric constant plot shifts towards higher frequencies.

**Fig. 9:**
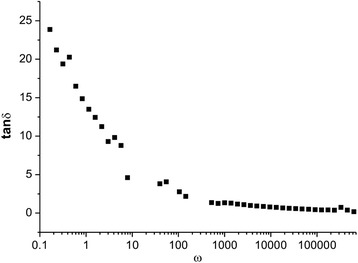
Dielectric tangents loss dependence at 273 K

On Fig. 9, there is a change in dielectric loss tangent (tanδ) at room temperature as a function of frequency.

Frequency behavior tanδ is typical for ferrites [6], its value decreases with the increasing frequency and its rate of decrease is greater at low frequencies, at high frequencies tanδ remains almost constant and amounts to 0.1–0.5 order value at frequencies >100 Hz. Dielectric loss tangent depends on several factors: stoichiometry, ion concentration Fe^3+^, structural uniformity, depending on the method of sample preparation and so on [5, 7]. Since the microstructure of fine material, which in synthesized system is a combination of grains, grain boundaries and structural defects, such samples generally show a high impedance at low frequencies due to contribution to the resistance of grain boundaries and low impedance at high frequencies, which is dominant in contribution resistance in the grain, where electron transport is carried out by the jump between iron ions, which are in different valence states.

**Fig. 10:**
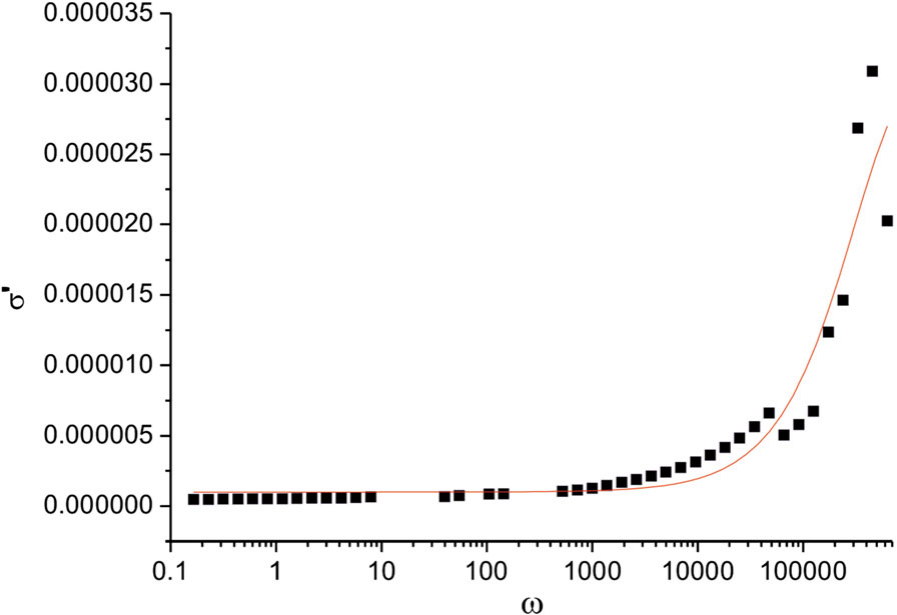
The dependence of the real part of the conductivity on frequency

Figure 10 presents frequency dependence of real part of conductivity.

At frequency *f* ≤ 10^4^Hz, *σ* is almost constant with frequency, but at *f* > 10^4^Hz, there is a change. Non-monotonously conductivity increases with the increase of frequency. Frequency dependence of AC conductivity can be explained basing on Kups theorem [15] according to which this system can be seen as a multilayer capacitor in which grains and grain boundaries have different properties. These characteristics show that above a certain frequency (*f* ~ 10^4^Hz), the effect of multilayer capacitor increases with the frequency, and as the result there is an increase in conductivity. Relaxation *σ* can be described from the perspective of relaxation formula [16]:

2 $$ \tilde{\sigma}\left(\omega \right)={\sigma}_{hf}+\frac{\sigma_{lf}-{\sigma}_{hf}}{1+{\left(\omega \tau \right)}^2} $$

where indexes l*f* and *hf* in the σ_lf_, σ_hf_ indicate the limit values σ at low (about 10^−2^Hz) and high (about 10^5^Hz) frequency, the relaxation time *τ* is a characteristic time constant of ferrites ω = 2πf.

The relaxation time τ for the synthesized system was calculated using the formula (5) and used to approximate experimental curve *σ*(*f*) (Fig. 8).

**Fig. 11:**
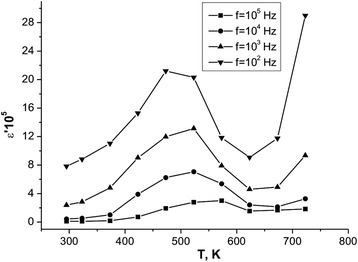
Temperature dependence of the real part of the dielectric constant of the system at different frequencies

Figure 11 shows the temperature dependence of the real part of the dielectric constant of the system at different frequencies.

You can see that the temperature dependence of the dielectric constant is characterized by a peak in the temperature range of 450–550 K, and with the increase of frequency there is a decreases of its intensity and the peak position shifts towards higher temperatures. The explanation of this dielectric anomalies as a function of temperature is based on the following factors: (1) thermally induced mechanism of electron hopping, (2) spatial charge polarization type of Maxwell-Wagner resulting in structural heterogeneity caused by the presence of a significant number of inter-grain borders that is the drain of uncompensated electric charges, and (3) a lithium oxygen vacancies arising due to partial loss of lithium in the fusion.

**Fig. 12:**
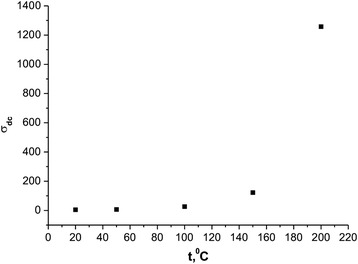
The temperature dependence of conductivity synthesized systems

The temperature dependence of conductivity of synthesized systems (Fig. 12) shows that it is inherent to the semiconductor conductivity, which is described by the relation type *σ* = *σ*_0_ exp[−*E*_0_/(*kT*)], where *σ*_0_ — approximated value of conductivity at *T* = 0, *E*_0_ — activation energy of electrical conductivity, *k* — the Boltzmann constant, *T* — absolute temperature.

**Fig. 13:**
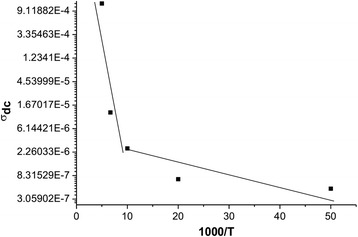
Dependence DC conductivity of inverse temperature (Arrenius plot)

In Arrhenius coordinates ln *σ*_*dc*_(10^3^/*T*) (Fig. 13), the temperature dependence of conductivity is well approximated at high (523–773 K) and low (295–373 K) temperatures, which is the evidence of activation of display and hopping conduction mechanisms, which are characterized by the growth of the conductivity with temperature (from sample slope approximating line). A different angle of approximating direct temperature in these areas suggests differences in the activation energy of electrical conductivity.

The calculated values ΔE of activation energy of high and low range is 2.46 and 1.42 eV, respectively. In the ambient temperature there is a dominant conduction mechanism of hopping is realized by electrons jump between the iron atoms that can be in different valence states.

According to X-ray analysis of long jump for tetrahedral (d_A_) and octahedral (d_B_) positions that were at ratios

3 $$ \begin{array}{c}\hfill {d}_A=0.25 a\sqrt{3},\hfill \\ {}\hfill {d}_B=0.25 a\sqrt{2}\hfill \end{array} $$

respectively was 3,610 ± 0.002 Ǻ and 2950 ± 0.002 Ǻ. Because tetrahedrical-sub-lattice is unlikely to present ions Fe^2+^, such electron migration is mainly in octahedrical-sub-lattice, as well as grain boundaries, which may focus on a significant amount of structural defects.

## Conclusions

There is sol–gel auto-combustion method synthesized by single-phase magnesium-substituted lithium ferrite spinel structure with space group Fd3m and a particle size of the order of 30–40 nm.

The main contribution to the dielectric dispersion of synthesized system and dipole is made in between limiting polarization. The high value of the dielectric constant at low frequencies at room temperature results from the exchange of electrons between the iron ions on the mechanism *Fe* ^2 +^ ⇔ *Fe* ^3 +^ and significant influence of inter-grain borders.

The conductivity of the synthesis has semiconductor character. There are two mechanisms of conductivity: activating—at high temperatures and hopping—at room temperature. Energy activation of both mechanisms significantly differ from each other, and constitute 2.46 and 1.42 eV for activating and hopping ones, respectively. The migration of electrons in the conduction mechanism of hopping is carried out within octahedrical-position long jump ~2.950 ± 0.002 Ǻ.

## Abbreviations

CRD: Region of coherent scattering
